# Oesophageal injuries: Position paper, WSES, 2013

**DOI:** 10.1186/1749-7922-9-9

**Published:** 2014-01-21

**Authors:** Rao R Ivatury, Frederick A Moore, Walter Biffl, Ari Leppeniemi, Luca Ansaloni, Fausto Catena, Andrew Peitzman, Ernest E Moore

**Affiliations:** 1 Surgery, Virginia Commonwealth University Health System, Richmond, VA, USA; 2 Acute Care Surgery, University of Florida, Gainesville, FL, USA; 3 Surgery, Denver Health Medical Center, University of Colorado School of Medicine, 777 Bannock St., MC 0206, Denver, CO 80204, USA; 4 Surgery, Meilahti Hospital, Haartmaninkatu 4, P.O. Box: 34000029 Helsinki, Finland; 5 Surgery I, Papa Giovanni XXIII Hospital, Piazza OMS 1, 24127 Bergamo, Italy; 6 Department of Emergency Surgery, Parma University Hospital, Parma, Italy; 7 Department of CSurgery, University of Pittsburgh, Pittsburgh, USA; 8 University of Colorado Denver, Denver, CO 80206, USA

## Abstract

The oesophagus is a difficult challenge for the surgeon because of its lack of serosal covering, the tenuous, segmental blood supply and the common delay in the diagnosis of injury. Early diagnosis is the key to successful management. Recent introduction of newer, minimally invasive techniques have provided management alternatives for both the normal and the diseased organ that is injured with both early and delayed diagnosis.

## Surgical anatomy

The oesophagus is a long, muscular organ that begins at the pharyngooesophageal junction at the level of the sixth cervical vertebra. It ends at the gastrooesophageal junction. The area of its origin at the cricopharyngeus muscle is an area of potential injury by the endoscopist or the neophyte anesthesiologist. Passing into the thorax, the oesophagus and the trachea traverse the superior mediastinum behind the great vessels and with a slight curve passes behind the left mainstem bronchus. From this point, the oesophagus curves to the right in the posterior mediastinum, curves back to the left behind the pericardium and crosses the thoracic aorta. Lying anterior to the thoracic aorta, it reaches the abdomen through the oesophageal hiatus of the diaphragm. There is no serosal covering for the structure. The outer layers are composed entirely of longitudinal and circular muscle fibers with squamous epithelium as the mucosal lining. The blood supply is segmental and is derived from branches of the inferior thyroid, bronchial, intercostal arteries and the aorta. Venous drainage is through submucosal channels into a perioesophageal plexus which eventually enters into the inferior thyroid and vertebral veins in the neck, the azygos and hemiazygos veins in the thorax and the left gastric vein in the abdomen.

## Introduction

Oesophageal perforation is a potentially life-threatening clinical situation with a high morbidity and a mortality. The clinical symptoms and signs are non-specific. The relative paucity of experience at any given center makes the diagnosis difficult and often delayed. There are no randomized studies, no class I evidence for diagnostic and management precepts. However, multiple series reported in the literature allow some strong recommendations.

## Review of literature

Oesophageal perforation is slightly more common in males [1-7] in their sixties.

Iatrogenic perforation is the most common cause of injury. The incidence is small, less than 0.5%, when all the procedures on the oesophagus are considered. Sclerotherapy of oesophageal varices, nasogastric tubes and improperly placed Sengstaken- Blakemore tubes have been known to produce oesophageal perforation. Oesophageal “stents”, temperature probes, repeated attempts at endotracheal intubation, impacted foreign bodies, both sharp and blunt, may all cause oesophageal injury. Blast injury and spontaneous rupture of the oesophagus are secondary to a sudden rise in intraluminal pressure and occur usually at the lower end of the oesophagus. Oesophageal trauma has been reported as a complication following anti-reflux procedures, pneumonectomy, truncal vagotomy (an incidence of 0.5%) and rarely, during anterior cervical spinal fusion Blunt oesophageal injury is exceedingly rare and often is missed. The predominant site of rupture is in the cervical and upper thoracic location (82.3%), and associated tracheooesophageal fistulas were noted in 28 patients in one series. Penetrating objects, usually GSW, injure the oesophagus more commonly than does blunt mechanism. It is not a very frequent injury. In a large multi-center study from the AAST, Asensio [3] collected 405 patients from 34 trauma centers over 10.5 years. Ingestion injury to the oesophagus may occur with caustic liquids [8], especially in children by cleaners, battery liquids and solutions used in industrial operations. Acids cause coagulative tissue necrosis with a lower risk of penetration while alkalis tend to be more palatable and cause liquefactive necrosis that rapidly becomes transmural. The amount, viscosity and concentration of the agent and the duration of contact between the caustic agent and the oesophageal mucosa determine the depth and extent of the injury.

## Diagnosis

The clinical symptomatology is non-specific early after perforation. Radiologic clues are subtle and may easily be missed. Consequently, delayed diagnosis of oesophageal perforation is extremely frequent. This is especially true in non-endoscopic iatrogenic trauma and after spontaneous perforation. In the AAST study [3], delayed diagnosis after penetrating trauma occurred in about 50% of patients reaching the operating room.

Pain, usually located in the chest with cervical perforations and perhaps referred to the abdomen with thoracic perforations, is a frequent complaint by patients with oesophageal perforation, occurring in 70% to 90% of patients. Pain preceded by repeated episodes of vomiting is a particularly important history that needs to be elicited. Dyspnea is the second common symptom, especially with thoracic perforations and infrequently is seen with cervical or abdominal perforations. Subcutaneous emphysema and crepitus are seen frequently with cervical perforations. Dysphonia, hoarseness, cervical dysphagia and subcutaneous emphysema are encountered in various combinations in this group of patients. There is sometimes acute abdominal or epigastric pain in patients with perforation of the gastro oesophageal junction. Notably, perforations rarely manifest with hematemesis or other signs of gastrointestinal bleeding, including melena [1-7].

## Plain radiographs

The radiologic findings that are suggestive of the diagnosis are free air in the soft tissues of the neck, and retropharyngeal or retro tracheal swelling. Chest radiographs may reveal free mediastinal or cervical air, mediastinal widening, pneumothorax, or, in delayed cases, pulmonary infiltrates.

## Contrast studies

Contrast oesophagography is indicated to confirm the diagnosis, localize the site of perforation and define the presence or absence of associated oesophageal pathology. In combined oesophageal and tracheal injuries or where there is suspicion of an abnormal oesophago-tracheobronchial communication, thin barium is the agent of choice. Free perforations into the pleura or the mediastinum (the presence of pneumomediastinum or pneumothorax) are best demonstrated by gastrografin. Once a gross extravasation is ruled out, a fluoroscopic study with thin barium is the next step to rule out a small perforation that may have been overlooked by the gastrografin study [1,2].

## Endoscopy

Endoscopy has a limited application as the *only* investigation. In instances of blunt or penetrating trauma where the patient is rushed to the operating room for control of other injuries, intraoperative oesophagoscopy may be employed to rule out gross oesophageal injury. Subtle perforations may be missed, especially by flexible endoscopy. In patients with a suspicion of oesophageal injury after external trauma, triple endoscopy (laryngoscopy, oesophagoscopy and bronchoscopy) is indicated. Injury to one of these structures should raise the suspicion of injury to the adjacent organs. The same principles are recommended for transmediastinal missile wounds as well as cervical penetrating wounds. The sensitivity and specificity of endoscopy in the diagnosis of oesophageal injury are unknown, but definitely are related to operator experience. The combination of contrast studies and endoscopy are accurate in more than 90% of patients. Intra-operative endoscopy while palpating the esophagus near the penetrating tract and insufflation of air looking for air-leak are useful techniques. Perforations caused by the endoscopist during oesophagoscopy are usually promptly suspected.

## Miscellaneous diagnostic methods

CT, in addition, may show collection of air or fluid in the mediastinum, pleural effusions, pneumopericardium and pneumoperitoneum as important diagnostic findings in these patients. The tract of the bullet in proximity to the esophagus gives another clue. The site of perforation and the degree of containment may also be noted. Tube thoracostomy for a hydrothorax with the demonstration of a continuous air leak not in synchrony with respiration may suggest an oesophageal injury. Increased levels of amylase in chest tube fluid in the appropriate clinical scenario is highly suggestive of oesophageal perforation [1-7]. Operative exploration is a useful diagnostic modality. Especially in patients with pressing indications for surgical exploration (hemorrhage, vascular injury), the oesophagus must be inspected in proximity injuries and operatively explored in the region of the penetrating wound. Adjunctive methods at exploration include instillation of saline or dye (methylene blue) intraluminally with manual compression of the organ to exclude a leak. The same purpose may be achieved by filling the operative field with saline and vigorously injecting air into the oesophagus to demonstrate an air leak. As mentioned earlier, intra-operative endoscopy is a useful option.

## Management

The choice of approach depends on the following factors: 1. the anatomic location of the perforation, 2. the time interval between the onset of perforation and the initiation of treatment, 3. whether the injury is contained or free, 4. the severity of illness of the patient, 5. the mechanism of injury and 6. Whether the oesophagus is normal or there is an associated lesion [1,3,5,6].

## Injuries to the cervical oesophagus

The management of cervical oesophageal perforation depends on the mechanism of injury. Neck exploration is performed through a left neck incision along the anterior border of the sternocleidomastoid muscle with medial retraction of the carotid vessels. Adequate mobilization behind the trachea and palpation of the nasogastric tube facilitate identification of the oesophagus. The recurrent laryngeal nerve needs to be protected in the dissection and frequently may be palpated or visualized. The oesophageal perforation is identified either by direct visualization or with the help of intraluminal saline or dye. The perforation is repaired in one or two layers. Neither the number of suture layers nor the type of suture material (absorbable or non-absorbable) seem to influence the incidence of fistulization after the repair. If the operative exploration is delayed, suturing may be difficult because of extensive inflammation in the area. In either instance (early or delayed operation), wide drainage is the key to success. Closed suction drains (Jackson- Pratt) usually are preferred. Broad-spectrum antibiotics (usually a synthetic penicillin) are commenced and continued peri-operatively. The drains are left for a period of 5–7 days. Most surgeons recommend a contrast study before the removal of the drain, because of the frequent occurrence of fistula without clinical symptomatology. Nutritional support may be delivered during this period by a nasogastric tube.

Cervical oesophageal fistulas are reported in 10% to 28% of cases after oesophageal repair. The factors that contribute to this complication include inadequate debridement, oesophageal devascularization, tension on the suture line and associated infection. Adequate drainage, exclusion of distal obstruction and maintenance of nutritional support are the cornerstones of fistula management and the majority of them heal with time [1,5].

Combined tracheo-oesophageal injuries: Combined tracheo-oesophageal trauma poses special problems: they are distinctly uncommon and thus may lead to management errors, they produce unique technical problems and may lead to complex complications in the remote postoperative period. Nearly always due to gun-shot injury, energy transfer; e.g., close range SGW vs. jacketed 32 caliber bullets determines the outcome. Feliciano and colleagues [3], based on an 11-year experience of 23 patients, recommend the following principles: 1. the addition of tracheostomy to a simple repair of the trachea may actually lead to a higher infectious morbidity in terms of pneumonia, mediastinal abscesses and wound infections. 2. For extensive oesophageal injuries in the cervical area, a cervical oesophagostomy, side or end, should be considered at the initial operation. 3. Sternocleidomastoid or, preferably, strap muscle interposition should be employed between tracheal and oesophageal repairs as well as to cover carotid artery repairs. It must be remembered that the sternocleidomastoid has a segmental blood supply in thirds and the upper (from occipital artery) and the middle (from the superior thyroid artery) are more reliable for flap creation. And 4. Drainage of combined cervical injuries should be directed anteriorly and through the contralateral neck if a carotid artery injury is present.

## Injuries to the thoracic oesophagus

### Iatrogenic and trauma related perforations

*Non-operative management:* A conservative, non-surgical approach occasionally is recommended for thoracic oesophageal perforations in selected patients. The perforation has to be contained for eligibility for non-operative management. Santos and Frater [8] described a system of “transoesophageal irrigation of the mediastinum” as a method of conservative management in patients with a delayed diagnosis of spontaneous rupture. The authors reported excellent results (7 of 8 survived) with a Levin tube placed in the oesophagus proximal to the tear, a chest-tube placed in proximity to the oesophagus, constant irrigation through the Levin tube and continuous suction to the chest tube: a method that ensured constant, mediastinal irrigation. Others used mediastinal irrigation by a transnasal catheter. Percutaneous drainage of pleural effusions, collections or abscesses [9], temporary endoscopic oesophageal stents [10-12] to seal oesophageal leakage and to recover gastrointestinal continuity are being recommended in selected patients. Use of endoscopic clips for perforation closure, endoscopic vacuum sponge therapy are being introduced recently to aid successful drainage and healing of oesophageal perforation or anastomotic insufficiency [2].

For instance, Fischer [13] reported in 2006 nonoperative treatment of 15 benign oesophageal perforations after endoscopic procedures with self-expandable covered metal stents. Seven patients (group 1) underwent stent insertion with an average time delay of 45 minutes. In 8 patients (group 2), the median delay was 123 hours. All patients in group 1 had an uneventful recovery and left hospital 5 days (range, 3 to 9) after stent insertion. One patient in group 2 (1 of 8) died of pneumonia after 6 days. In the other 7 cases, perforations healed successfully after stent placement, but the clinical course was generally complicated with sepsis and multiple organ failure. The average hospital stay was 44 days (range, 15 to 70).

Linden [9] described 43 procedures on the oesophagus with a 30-day or in-hospital mortality of 7.0% and an overall morbidity of 47%. Most acute thoracic oesophageal perforations were treated with primary repair with a low mortality rate of 5%. Most delayed perforations were treated with T-tube repair and had a mortality rate of 8.7%. The complication rate was much lower in the in the group repaired within 24 hours.

Freeman [10] reported on 17 patients treated with silicone-coated stents placed endoscopically utilizing general anesthesia and fluoroscopy with adequate drainage of infected areas. Leak occlusion was confirmed by oesophagogram in 16 patients (94%). Fourteen patients (82%) were able to initiate oral nutrition within 72 hours of stent placement. One patient (6%) experienced a continued leak after stent placement and underwent operative repair. Stent migration requiring repositioning (2) or replacement (2) occurred in 3 patients (18%). All stents were removed at a mean of 52 +/− 20 days after placement. Hospital length of stay for patients treated with oesophageal stent placement was 8 +/− 9 days (median, 5). In another variation of non-operative treatment, Linden [9] used T-tube repair in delayed perforations with a mortality rate of 8.7%. In another recent series (12), 14 consecutive patients with spontaneous oesophageal perforation were treated with coated self-expandable stent and a debridement procedure (three patients by thoracotomy, four by thoracoscopy, three by tube drainage, and two patients with no drainage). Eight patients had one stent, while six patients needed one or more additional stents to achieve source control. Two patients (14%) died during the in-hospital stay, both of them having received more than one stent. Eight patients had one stent, while six patients needed one or more additional stents to achieve source control. Fourteen percent of patients who underwent stenting within 24 hours to stent placement were in septic shock compared with 86% of patients with a delay of more than 24 hours.

In a recent review, Kuppusamy [11] described 81 consecutive patients with acute oesophageal perforation. 48 patients (59%) were managed operatively, 33 (41%) nonoperatively, and 10 patients with hybrid approaches involving a combination of surgical and interventional techniques; 57 patients (70%) were treated <24 hours and 24 (30%) received treatment >24 hours after perforation. LOS was lower in the early-treatment group; however, there was no difference in complications or mortality. Nonoperative therapy increased from 0% to 75% over time. Nonsurgical therapy was more common in referred cases (48% vs 30%) and in the >24 hours treatment group (46% vs 38%). Over the period of study, there were decreases in complications (50% to 33%) and LOS (18.5 to 8.5 days). Mortality for the entire series involved 3 patients (4%): 2 operative and 1 nonoperative. The author concluded that referral to a tertiary care center, treatment within 24 hours, an experienced surgical management team using a diversified approach can expect to shorten LOS and limit complications and mortality.

Surgical intervention is indicated if the patient should worsen on conservative treatment or should develop a mediastinal abscess or empyema. The presence or the development of pneumothorax, pneumoperitoneum, systemic signs of sepsis or shock are contraindications for a nonoperative approach. Non-operative treatment should also be used when the perforation is related to an inoperable malignant stricture. Patient outcome depends mainly on the proper treatment of mediastinal and pleural contamination. Indications for percutaneous drainage or more extensive drainage by surgical intervention should be considered carefully if there is gross contamination [1,11].

Operative management: Operative repair is the treatment of choice for free perforations. This is true for injuries diagnosed both early (< 24 hours) and late (> 24 hours.) The operative approach consists of thoracotomy on the side of the leak (left thoracotomy for lower oesophageal injury and right thoracotomy for upper oesophageal injury), exposure of the oesophagus and thorough debridement of all necrotic tissue. The perforation is identified and closed. In penetrating trauma, multiple perforation are not uncommon and should be looked for diligently. The choice of suture material for closure of the perforation is variable between surgeons, as is the necessity for a two-layered closure with an inner absorbable and outer nonabsorbable sutures. A pleural flap or various neighboring structures (diaphragm, intercostal muscle, vascularized or a free graft of pericardium, extracostal chest wall muscle, omentum or a pedicled jejunal segment) may be used as a “buttress” to the repair… In the lower thoracic area, the gastric fundus has been used as an onlay type of patch by enlarging the oesophageal hiatus and bringing the gastric fundus to the perforation. Drainage of the area extensively, usually with large caliber chest tubes placed in the vicinity of the oesophageal repair, is the most important part of treatment. Primary repair of oesophageal perforation is possible, especially in patients admitted to the hospital within 24 hours of the event. However, multiple recent studies found that mortality risk was not related to wait time exceeding 24 Hours. When repair is attempted in iatrogenic cases with a stricture distal to the perforation, a myotomy might be indicated and the defect covered with a fundoplication. Repair over a T-tube is an alternative treatment that allows for a controlled esophago-cutaneous fistula to be established. This allows healing to take place without contamination [9]. The T-tube can be removed in most patients after 4–6 weeks, and the fistula will eventually close.

With recent advances in video endoscopy, identification and repair of oesophageal perforation by Video Assisted Thoracic Surgery (VATS) has been reported. The future will determine if this modality will enable an earlier, more efficient recognition of oesophageal injury.

Treatment of delayed recognition of the perforation: Oesophageal exclusion and other adjunctive techniques: The problems of delayed treatment involve extensive mediastinitis, necrosis of the oesophageal wall and the difficulty of effectively closing the perforation, even with various buttressing methods. Even when repair is technically feasible, subsequent breakdown of the repair is the rule rather than the exception. It is in such patients that “exclusion” procedures were previously recommended. The rationale for this approach is to exclude the repair from the rest of the oesophagus and allow it to heal while nutritional support is maintained by intravenous or enteral route. The decision to perform exclusion or repair depends on the local findings at thoracotomy as well as the time delay between perforation and operative treatment. In several series, exclusion procedures generally were reserved for a delay in treatment of more than 48 hours.

The principles of exclusion procedures are: 1. to divert the oesophagus from above, 2. to prevent gastric reflux from below and 3. To drain the area widely, usually by tube thoracostomy and 4. Feeding jejunostomy.

1. Diversion from above: by a long T-Tube with the side arm brought out through the perforation and the chest wall to divert the saliva and achieve a controlled fistula. Other techniques described included a lateral cervical oesophagostomy by making an opening in the cervical oesophagus and suturing the opening to the skin. The oesophagus distal to the ostomy may be closed or stapled. 2. Diversion from below: Some authors recommended looping the distal oesophagus with a prolene suture that is brought out of the abdomen along with a gastrostomy. After the oesophageal perforation healed, the Prolene suture was removed, without laparotomy, restoring oesophageal continuity [14].

The problem of exclusion-diversion procedures is that the majority of these patients require a secondary procedure to restore continuity of the GI tract after the fistula had healed. These procedures involve a colon or gastric interposition, depending on the surgeon’s preference. In many instances, the exclusion becomes permanent. Oesophageal exclusion is now reserved for the very poor risk patient who cannot tolerate any major surgical procedures.

Perforation with pre-existing pathology: *Oesophageal Resection:* Emergency resection of the perforated oesophagus is undoubtedly the treatment of choice when there is associated distal obstruction. The results of oesophagectomy for simple or delayed perforations with or without associated oesophageal disease have been poor in most series. A more optimistic evaluation of emergency oesophagectomy for oesophageal disruption was reported by Orringer and Stirling [15]. A diverse group of 24 patients was presented including 20 with preexisting oesophageal diseases (chronic strictures, achalasia, reflux esophagitis, carcinoma, diffuse oesophageal spasm and monilial esophagitis). Forty-five percent of the patients had a delay of > 3 days prior to oesophagectomy. Alimentary tract continuity was restored in 13 of the 24 by oesophagogastric anastomosis. In 11 patients, the oesophagus was resected preserving as much of the normal esophagus as possible. The proximal oesophagus was then delivered into the neck, tunnelled in front of the clavicle and the end was constructed as an ostomy on the chest wall. The authors felt that the risk of oesophageal resection in these patients was less than that from repair or exclusion procedures.

Recent series of oesophageal injury: Eroglu [16] performed a retrospective clinical review of 44 patients treated for oesophageal perforation in 2009. Perforation occurred in the cervical oesophagus in 14 patients (32%), thoracic oesophagus in 18 patients (40%), and abdominal oesophagus in 12 patients (27%). The perforation was treated by primary closure in 23 patients (52%), resection in 7 patients (16%), and nonsurgical therapy in 14 patients (32%). In the surgically treated group, the mortality rate was 3 of 30 patients (10%). 2 of 14 patients (14.3%) died in the conservatively managed group. Four of the 14 nonsurgical patients were inserted with covered self-expandable stents. Describing a single surgeon experience, Kiernan et al. [17] reported on 48 patients with a survival of 96% with early surgical treatment. Even when the diagnosis was delayed > 24 hours, hospital survival was 82.6%, increasing to 92.3% when treated with surgery. The authors recommended aggressive, definitive surgery for thoracic oesophageal perforations and reserved conservative, medical therapy in patients with ‘microperforations’ with no continuing leak.

Richardson [18] summarized the results of aggressive surgical management for oesophageal perforation. All were treated by operative repairs, buttressed with muscle or pleura. Sternocleidomastoid muscle was used to buttress or primarily close the defects in the neck, and a flap of diaphragm was often used for thoracic perforation. Patients with perforated cancer or severe underlying disease had an oesophagectomy. With these techniques, 50 of 64 patients underwent preservation of the oesophagus after closure of the perforation and 14 underwent resection. The leak rate was 17%, but all healed. One patient treated with primary closure died (1.5% mortality) and only 1 patient required subsequent oesophagectomy.

Vallböhmer [19] described an institutional experience of 44 patients over a period of 12 years. Iatrogenic injury was the most frequent cause of oesophageal perforation. Eight patients (18%) underwent conservative treatment with cessation of oral intake, antibiotics, and parenteral nutrition. Twelve (27%) patients received an endoscopic stent implantation. Surgical therapy was performed in 24 (55%) patients with suturing of the lesion in nine patients, oesophagectomy with delayed reconstruction in 14 patients, and resection of the distal oesophagus and gastrectomy in one patient. The hospital mortality rate was 6.8% (3 of 44 patients): one patient with an iatrogenic perforation after conservative treatment, and two patients after surgery (one with Boerhaave syndrome, one with iatrogenic rupture). No death occurred in the 25 patients when the diagnosis was made in less than 24 hours. When it was delayed, 19% of 16 patients died (P = 0.05).

Keeling et al. [20] in 2010 retrospectively reviewed all cases of oesophageal perforation from 1997 through 2008 at Emory University. Among 91 patients, the perforation was iatrogenic in 50 (52%), spontaneous in 23 (24%), and idiopathic in 22 (23%). The authors concluded that the overall mortality from oesophageal perforation can be less than 10%. Primary repair should be considered as first-line treatment when appropriate even in patients who present more than 24 hours after perforation. Non- operative management, in appropriate patients, can be used in selected patients. Similar results were recorded by the Houston group [21] and two recent meta-analyses [22,23].

## Results and prognostic considerations

In the multi-institutional series reported by Asensio [4], a logistic regression of 346 patients reaching the O.R. after penetrating trauma established that a delay in preoperative evaluation, AAST organ injury score > 2 and resection and diversion were independent factors for increased oesophagus-related complications. The prognosis appears to be much improved with modern approaches to diagnosis and critical care but is still high with delayed diagnosis and treatment. Emphasis should be placed on early diagnosis of injury and careful selection of operative versus non-operative treatment by experienced clinicians. The excellent results with nonoperative management of iatrogenic injuries mask the potential life-threatening complications of pathologic lesions, and trauma is in between.

## Recommendations

We recommend a strong suspicion for oesophageal injury in the appropriate clinical situation of potential injury to the organ and aggressive pursuit of diagnosis to be made within 12 to 24 hours. CT scanning is a useful diagnostic modality in cases of suspected perforation.

We recommend prompt surgical exposure and closure of oesophageal perforation in layers with adequate drainage of the area and antibiotic therapy. In cervical oesophageal injuries with associated tracheal or vascular repairs, these should be separated from the oesophageal repair by sternocleidomastoid or strap muscle interposition.

We recommend that the treatment of the injured oesophagus be given by clinicians experienced in the endoscopic or surgical management of the organ, ideally in a tertiary center with multispecialty availability by experienced clinicians.

We suggest non-operative management of small perforations diagnosed within 24–48 hours in a stable patient with no mediastinitis or empyema.

In non-trauma injuries, that are initially missed and/or present in a delayed fashion, the initial management of sepsis by resuscitation, antibiotics and chest drainage is the priority. A variety of techniques including stents, t-tubes and clipping are available and should be individualized to the clinical situation and patient. These patients need nutritional supplementation, preferably enteral, while the oesophagus heals. We suggest careful observation of these patients for signs of escalating septic complications and prompt surgical intervention, should these occur.

We suggest oesophageal resection by experienced surgeons for perforation of the diseased organ and planned reconstruction of esophago-gastric continuity.

## Competing interests

The authors declare that they have no competing interests.

## Authors’ contributions

“RRI drafted the manuscript. FAM, WB, AL, LA, FC, AP, EEM reviewed the draft and made corrections and revisions”. All authors read and approved the final manuscript.
